# Botulinum Toxin Type A and the Prevention of Hypertrophic Scars on the Maxillofacial Area and Neck: A Meta-Analysis of Randomized Controlled Trials

**DOI:** 10.1371/journal.pone.0151627

**Published:** 2016-03-17

**Authors:** Dai-zun Zhang, Xiao-ya Liu, Wen-lin Xiao, Yao-xiang Xu

**Affiliations:** 1 Department of Stomatology, the Affiliated Hospital of Qingdao University, Qingdao, 266003, China; 2 The Key Laboratory of Oral Clinical Medicine of Shandong Province, the Affiliated Hospital of Qingdao University, Qingdao, 266003, China; Institute Pasteur, FRANCE

## Abstract

**Background:**

The purpose of the meta-analysis was to evaluate the efficiency of therapeutic botulinum toxin type A (BTX-A) in the prevention of maxillofacial and neck scars.

**Methods and Findings:**

Information came from the following electronic databases: Medline, PubMed, Cochrane Library, and EMBASE (time was ended by August 31, 2015) to retrieve RCTs evaluating the effect of the BTX-A for hypertrophic scar on the maxillofacial or neck. All languages were included as long as they met the inclusion criteria. Here the effects of BTX-A were evaluated by comparing the width of the scar, patient satisfaction, and the visual analysis scores (VAS), respectively. Pooled weighted mean differences (WMDs), pooled odds ratios (ORs), and 95% confidence intervals (CI) were calculated. Nine RCTs covering a total of 539 patients were included. A statistically significant difference in scar width was identified between the BTX-A group and control group (non-BTX-A used) (WMD = -0.41, 95% CI = -0.68 to -0.14, *P* = 0.003). A statistically significant difference in patient satisfaction was observed between the BTX-A group and control group (OR = 25.76, 95% CI = 2.58 to 256.67, *P* = 0.006). And in patients regarding visual analysis scores (VAS), a statistically significant difference was also observed between the BTX-A group and control group (WMD = 1.30, 95% CI = 1.00 to 1.60, *P* < 0.00001).

**Conclusions:**

This meta-analysis evaluates the efficacy of the BTX-A and confirms that BTX-A is a suitable potential therapy for the prevention of hypertrophic scars in patients in the maxillofacial and neck areas.

## Introduction

Patients are often disappointed if they have an ugly scar especially a hypertrophic scar on their face or neck [1]. In both western and eastern societies, hypertrophic scarring is often regarded as aesthetically displeasing [2]. It troubles many people by restricting the movements of their joints in one or more directions, reducing the functional performance of the face, which, given the role of facial expressions in everyday human interaction, can bring considerable physical and psychological harm [3]. Currently, there is no effective method of eliminating scars completely, probably due to the limited understanding of the complex mechanisms underlying the process of excessive scarring [4]. A great deal of literature has proved that, during the wound healing process, tension exerted on the wound edges is one of several important factors that can affect wound healing of cutaneous tissue [5]. Meanwhile, a broad body of literature has shown that BTX-A can lessen the intensity of tensile forces by inducing temporary paralysis of the muscles that injected into the wound edges [5–7].

The use of botulinum toxin to minimize facial scarring has a long history, going back to the early days of its discovery. BTX-A is a neurotoxin produced by bacteria. It has been utilized to induce chemo-denervation of muscles. It plays this role by inhibiting the release of acetylcholine at the neuromuscular junction [8]. In recent years, BTX-A has become a useful method of treating anorthopia, removing wrinkles, and minimizing hypertrophic scars on the body [9].

Hypertrophic scarring is the result of the excessive fibrosis during the process of wound healing [10]. It causes red, raised, and sometimes itchy scars at the site of the original surgical incision. The scars may grow rapidly for 3 to 6 months and then regress. They generally mature by increasing in width.

## Methods

### Search strategy

The Medline, Cochrane, Embase, and Google Scholar databases were searched using combinations of the following terms: “Botulinum toxin type A” or “BTA,” “skin scar,” “facial wound,” “oral and maxillofacial,” “neck.” The search was performed for studies published any time before August 31, 2015. After all the databases were searched, relevant studies, such as cited references, were hand-searched to indicate additional studies that might meet the inclusion criteria. Duplicate documents were excluded. A 2-step process was used to identify the final studies that would be included in the meta-analysis. First, the title and abstract of each article were screened, and citations not meeting the inclusion criteria were discarded. In the second step, two readers read the whole text of the remaining articles and made sure each one met all of the inclusion criteria. Studies were identified through the search strategy by 2 independent reviewers. If there were any uncertainties regarding eligibility, a third reviewer was consulted.

### Selection of studies

The following inclusion criteria were used for the meta-analysis:

1. The studies were RCTs.
2. The studies evaluated the effects of BTX-A on the oral, maxillofacial, or neck scars.
3. The related studies covered patients who had been diagnosed with hypertrophic scarring, including babies born with cleft lips who were slated for primary cheiloplasty, individuals (16 years or older) slated for revisional surgery due to unsightly outcomes of primary cheiloplasty, and individuals with facial wounds from injuries and other causes.
4. Interventions involved injection of BTX-A with normal saline as a control treatment, BTX-A and normal saline were injected alone and not combined with any other treatments. Outcomes of BTX-A and normal saline were compared.
5. The studies contained sufficient raw data for establishment of weighted mean difference (WMD) with 95% confidence intervals (CI).

The following exclusion criteria were used for the meta-analysis:

1. The study lacked sufficient raw data available;
2. It was a repeated or duplicate publication;
3. There were no usable data reported in literature.
4. The studies were conducted on animals, pregnant women, individuals planning to become pregnant or begin breast feeding, or patients with mental or nervous conditions.
5. The study covered keloids, burn scars, or participants who could not participate in the whole process.
6. The study covered people who were allergic to botulinum toxin or who had myasthenia, any previous injection of botulinum toxin within the 6 months prior to enrollment, or who refused to participate in the trial.
7. letters, case reports, comments, and editorials were also excluded.

The study was divided into several RCTs and the measurements were used separately if there were multiple measurements method in one study about the same assessment.

### Data extraction

Two reviewers extracted the data from eligible studies. In cases of disagreement, they consulted a third reviewer until the problems were resolved. Study characteristics and results were extracted in a standardized form which included the name of the first author, country, year of publication, duration of follow-up, number of participants in each treatment group, location of the scar, and observed indicators.

### Outcome measures

Observed indicators between BTX-A group and control group were re-extracted from all the selected eligible studies to make sure there were enough data to analyze. Final indicators were divided into 3 parts: photographic measurements of scar width, efficacy of BTX-A, and visual analogue scale (VAS). All the indicators were recorded by an experienced observer in an independent and blinded fashion. After the indicators were defined, the statistical significance of the findings was calculated.

### Statistical analysis

The differences in outcomes, which included scar width, patient satisfaction, and the outcome of VAS were compared between participants receiving either BTX-A or placebo therapy. The mean measure of the outcomes was expressed with weighted mean difference (WMD) or odds ratio (OR) with 95% confidence intervals (95% CI) and *P* < 0.05 was considered statistically significant.

Heterogeneity was examined using the Chi-square test. Random-effects models were used if heterogeneity was detected (I^2^>50%). Otherwise, fixed-effects models were used. Sensitivity analysis was performed using the leave-one-out approach. All analyses were performed with Comprehensive Meta-Analysis statistical software, version 5.3 (Copenhagen, Denmark), which was provided by the Cochrane Collaboration.

### Quality assessment

Quality of included studies was assessed and appraised for methodological quality using the Risk of Bias tool in Review Manager 5.3 (Copenhagen: The Nordic Cochrane Centre, Cochrane Collaboration, 2014) and by constructing a funnel plot.

## Results

### Study selection

**Fig 1:**
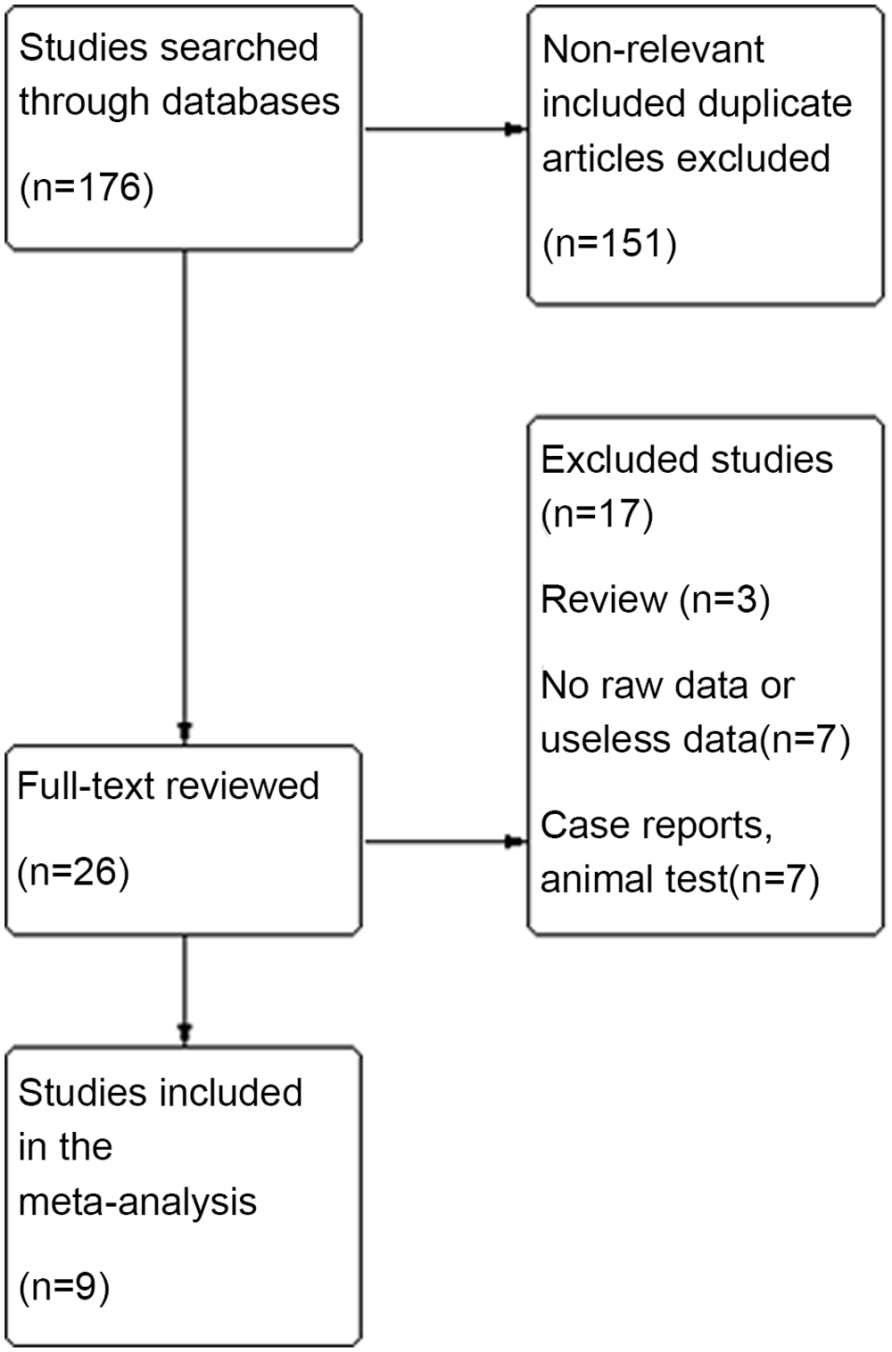
Flow diagram for study selection.

A flow diagram of study selection is shown in Fig 1. The initial database search yielded 176 studies. Of these, 26 underwent full-text review and 17 were excluded [6, 10–25]. Among these excluded studies, three were conducted on animals [11–13], four were case reports [14,16,18,21], three were reviews [6,10,17], and another seven lacked raw data or contained useless data [15,19–20,22–25] (S1 File). Finally, nine articles met the inclusion criteria and were included in the analysis [1, 26–33].

### Study characteristics

Study characteristics are shown in Table 1. The reasons for the 17 excluded studies are shown in Table 2. Of these nine included papers, there are 7 English publications and 2 Chinese publications, a total of 539 patients were included in the analysis. They came from Germany, France, Egypt, Korea, and China. There were three articles covering six RCTs that analyzed the width of the scar [26–28]. Collectively, there were 189 patients in the BTX-A groups and 184 controls. Whereas, the patients felt satisfaction in the study were four article and covering 172 people in the BTX-A group, 172 in the control group [29–32]. Two articles analyzed the score of VAS by using standard differences [26–27], and the other two articles used median VAS [1,33].

**Table 1:** Basic characteristic of included RCT studies in the Meta-analysis.

Studies (year)	Country	Follow up	BTA/Placebo	Scar location	Outcome indicators
Chang CS 2014^26^	Taiwan	6 mo	30/29	Upper lip	VSS, VAS, width of scar
Chang CS 2014^27^	Taiwan	6 mo	30/28	Upper lip	VSS, VAS, width of scar
Gassner HG 2006^1^	American	6 mo	16/15	forehead	median VAS
Ziade M 2013^33^	France	1 y	11/13	facial	MedianVAS,medianVSS,medianPSAS,median OSAS
Wilson AM 2006^29^	Egypt	1 y	40/40	facial	Patients’ satisfaction
Xiao Z 2009^30^	Chinese	0.5 y	3/3	face, neck	Patients’ satisfaction,erythema,pliability,and itching score
Kim YS 2014^31^	Korea	6 mo	15/15	neck	SBSES,Patients’ satisfaction
Wang XY 2013^32^	Chinese	1 y	114/114	facial	Patients’ satisfaction
Li WH 2014^28^	Chinese	1y	39/42	facial	Width of scar

BTA, BTA treatment group; Placebo, placebo treatment group; VSS, vancouver scar scale; VAS, visual analogue scale; median PSAS, median patient scar assessment scale; median OSAS,median observer scar assessment scale;SBSES, stony brook scar evaluation scale.

**Table 2:** Reasons for excluded studies.

Study	Reason
Al-Qattan MM^6^	Review
Berman B^10^	Review
Gassner HG^11^	Animal text
Lee BJ^12^	Animal text
Xiao Z^13^	Animal text
Uyesugi B^14^	Case report
Babuccu B^15^	No raw data/useless data
Goodman GJ^16^	Case report
Feily A^17^	Review
Gassner HG^18^	Case report
Jablonka EM^19^	No raw data/useless data
Laskawi R^20^	No raw data/useless data
Tollefson TT^21^	Case report
Flynn TC^22^	No raw data/useless data
Shaarawy E^23^	No raw data/useless data
Park TH^24^	No raw data/useless data
Robinson AJ^25^	No raw data/useless data

### Outcomes

**Fig 2:**
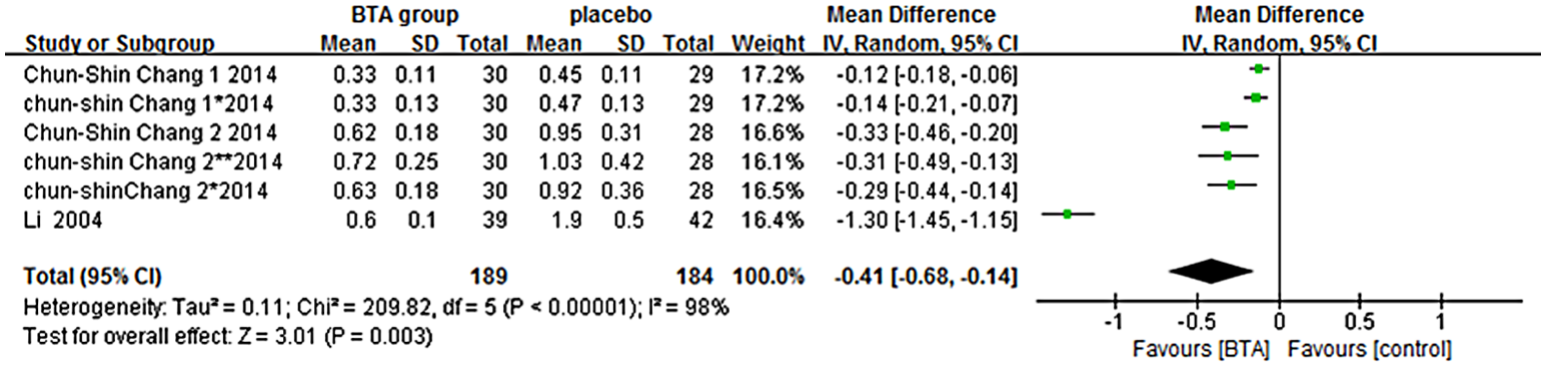
The width of scar forest plot (BTA vs. Placebo).

Width of scar: Only 3 articles were included in the analysis [26–28]. Because two articles demonstrated two points of scar width each, they were here regarded as four RCTs and brought into analysis [26–27]. To sum up, a random-effects model was used because there was evidence of heterogeneity among these studies (Chi^2^ = 209.82, *P* < 0.00001, I^2^ = 98%). The results showed the difference to be statistically significant (WMD = -0.41, 95% CI = -0.68 to -0.14, *P* = 0.003) (Fig 2), indicating that BTX-A was associated with narrower scars.

**Fig 3:**
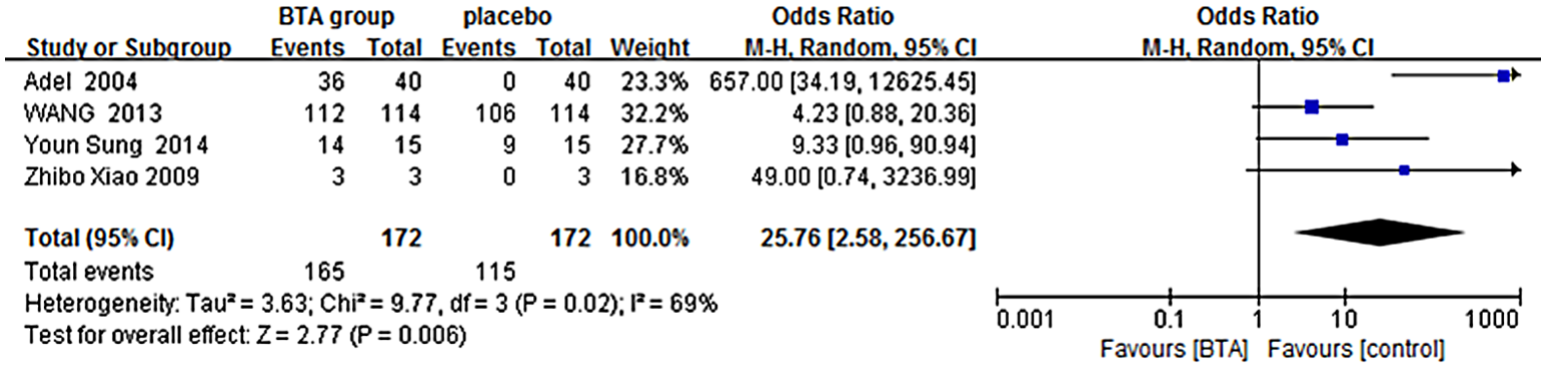
favorable vs. Unfavorable forest plot.

Patient satisfaction: A random-effects model was used because of the evidence of heterogeneity noted in this analysis (Chi^2^ = 9.77, *P* = 0.02, I^2^ = 69%). Results showed the difference to be statistically significant (OR = 25.76, 95% CI = 2.58 to 256.67, *P* = 0.006) (Fig 3), indicating that people who experienced hypertrophic scarring were quite satisfied with BTX-A therapy.

**Fig 4:**
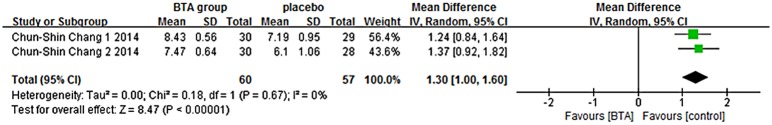
VAS forest plot (BTA vs. Placebo).

Score of scar: Evaluation of scarring was performed using a standard visual analogue scale (VAS). VAS is a subjective evaluation index that can indirectly reflect the effect of BTX-A and the scale graded from 0 (worst possible scar) to 10 (best possible scar) [26]. Four articles reported VAS [1,26–27,33]. Among them, two articles described VAS, and no heterogeneity was observed between them (Chi^2^ = 0.18, *P* = 0.67, I^2^ = 0%) (Fig 4), so the fixed- effects model was used [26–27]. Results showed the difference to be statistically significant (WMD = 1.30, 95% CI = 1.00 to1.60, *P* < 0.00001). The other two described the median VAS [1,33]. The average score was 8.9 in the BTX-A group and 7.2 in the placebo group in one study and 8.25 for the BTX-A group and 6.28 for the placebo group in the other. Based on these results, it was concluded that BTX-A had significantly more favorable results in the treatment of hypertrophic scarring than placebos.

### Quality assessment

**Fig 5:**
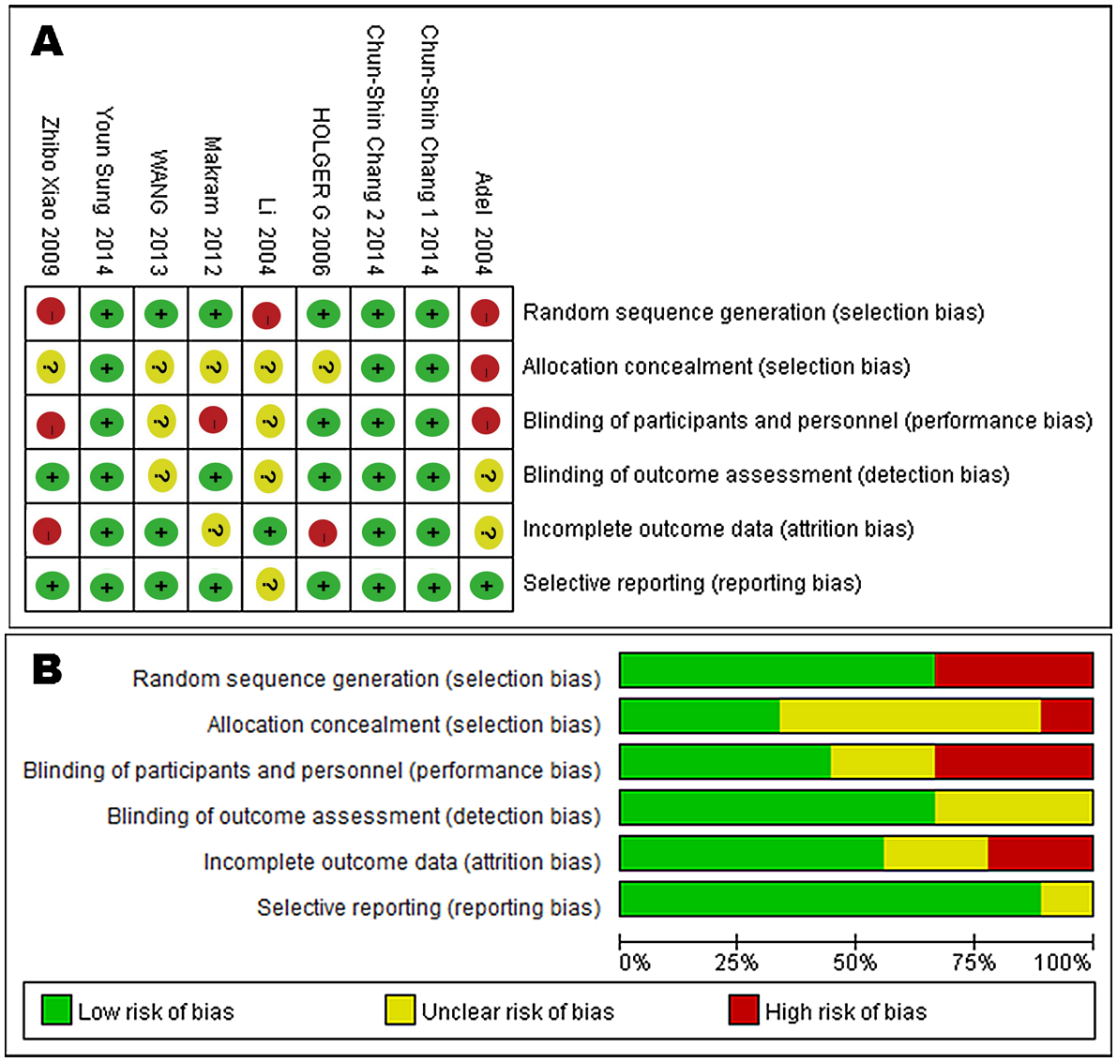
Quality assessment of each included study. (A) Risk of bias summary. (B) Risk of bias graph.

Results of the quality assessment of the nine trials are shown in Fig 5. Only three trials were unbiased in all six categories, and the others had a high risk of performance or detection bias.

### Sensitivity analysis

Sensitivity analysis was performed using a leave-one out approach; the clinical outcomes did not differ markedly upon the exclusion of any one individual study, indicating that the meta-analysis has strong reliability.

### Publication bias

Because of the limited number of studies in each outcome measurement in this meta-analysis, publication bias was not assessed with Begg’s funnel plot.

## Discussion

The management of hypertrophic scars has always been controversial. Maxillofacial and neck scars are often considered unattractive and have frustrating practical problems. They are the product of the excessive growth of benign fibers and they can cause functional and cosmetic deformities, psychological stress, pain, itching, and other uncomfortable symptoms [34]. Such deformities significantly reduce the quality of life and affect functional performance. It has been reported that most of recently investigated patients are pleased with even small improvements in scars [2]. Over the years, a great number of methods have been proposed to improve scars, such as BTX-A, pressure therapy, gross excision, laser therapy and vascular endothelial growth factor inhibitors and other methods [35]. However, no consensus has been reached regarding the best course of treatment due to the dearth of evidence-based information. An optimal management approach should be defined. This would resolve this problem.

In recent years, BTX-A has become more and more popular and it has seen use in a variety of indications in humans, including blepharospasm, spastic dysphonia, and hyperfunctional facial lines [36–38]. BTX-A is a potent neurotoxin created by *Clostridium botulinum*. It produces flaccid paralysis in striated muscle lasting about six months by inhibiting the release of acetylcholine at the neuro-muscular junction [8]. The advantages of the chemical agent could cause temporary denervation and could be useful in achieving the desired effect of decreasing muscle pulls.

Hypertrophic scars are often formed by high tension and stretching at the site of a wound site. Local movements of the body such as the upper and lower lip, cheek, forehead, and neck may cause muscle pulls at the edges of a wound [35]. It has been reported that the normal wound healing process can be divided into four different phases: hemostasis, inflammation, proliferation, and remodeling. Muscle pulls are believed to extend into the inflammatory phase during wound healing [3]. Investigating the effects of BTX-A could provide clinicians with a platform for solving the problem of hypertrophic scars and at improve patient outlook.

In the nine papers searched here, the width of the scar was consistently narrower in BTX-A groups than control groups (WMD = -0.41, 95% CI = -0.68 to -0.14, P = 0.003). Chang et al. and Li et al. analyzed data regarding scar width, and they found that the results in the BTX-A and control groups to be statistically significantly different [26–28]. In the other four papers, the authors analyzed patient satisfaction using subjective measurements [29–32]. Result showed that people who had hypertrophic scars also had a high assessment on the appearance outcomes. Wilson reported that 90% of patients were pleased with the narrower scars [29]. Few researchers found that the width was not significantly improved relative to the patient’s preoperative appearance. Xiao et al. studied BTX-A injection in people with hypertrophic scars by evaluating erythema, pliability, and itching sensations, they concluded that the outcome was excellent [30].

The data provided by this meta-analysis indicated that the use of BTX-A is safe and effective which was consistent with the results of trials conducted on primates by Gassner. In Gassner’s study, researchers investigated the use of BTX-A to improve forehead scars and found there to be a significant improvement in cosmetic appearance in the toxin group [11]. The studies listed above demonstrate a consistent beneficial effect of botulinum toxin in preventing facial and neck scars. Both yielded better cosmetic outcomes. So far, there has been no comprehensive review or meta-analysis about the effect of BTX-A. However, the present work does have limitations that should be carefully considered when interpreting the results. The analysis conducted here may not have taken the differences in patient ages into account; they ranged from three months to 70 years old. Second, the characteristics of patients in the included studies were not homogeneous. Third, only a few events were studied because of a lack of evidence illustrating the results. Lastly, several of the studies were found to have a high risk of performance or detection bias. These facts all showed that further research should be conducted in this field to provide additional high-quality studies of this issue.

## Conclusions

These results suggest that BTX-A is more effective and useful than non-BTX-A in eliminating hypertrophic scars from the maxillofacial area and neck. BTX-A could improve the quality of the scars and meet patients’ cosmetic requirements. However, because there were only a few studies, further clinical practice should be performed and larger databases should be consulted to better determine the efficiency of BTX-A.

## Supporting Information

S1 File Lists of full-text excluded articles. (DOCX)

S1 PRISMA Checklist PRISMA Checklist. (DOC)
