# Supplementary material for: Botulinum Toxin Type A and the Prevention of Hypertrophic Scars on the Maxillofacial Area and Neck: A Meta-Analysis of Randomized Controlled Trials
Source: PLoS One. 2016 Mar 17;11(3):e0151627. doi: 10.1371/journal.pone.0151627 (PMC4795777; doi:10.1371/journal.pone.0151627)
Supplement: S1 File — (DOCX) [file pone.0151627.s001.docx]

**The list of excluded articles**

1. Al-Qattan MM, Al-Shanawani BN, Alshomer F (2013) Botulinum toxin type A: implications in wound healing, facial cutaneous scarring, and cleft lip repair. Ann Saudi Med 33:482-488.

2. Berman B, Villa AM, Ramirez CC (2004) Novel opportunities in the treatment and prevention of scarring. J Cutan Med Surg 8:32-36.

3. Gassner HG, Sherris DA, Otley CC (2000) Treatment of facial wounds with botulinum toxin A improves cosmetic outcome in primates. Plast Reconstr Surg 105(6):1948-1953.

4. Lee BJ, Jeong JH, Wang SG, Lee JC, Goh EK, Kim HW (2009) Effect of botulinum toxin type a on a rat surgical wound model. Clin Exp Otorhinolaryngol 2(1):20-27.

5. Xiao Z, Qu G (2012) Effects of botulinum toxin type a on collagen deposition in hypertrophic scars. Molecules 17(2):2169-2177.

6. Uyesugi B, Lippincott B, Dave S (2010) Treatment of a painful keloid with botulinum toxin type A. Am J Phys Med Rehabil 89(2):153-155.

7. Babuccu B, Babuccu O, Yurdakan G, Ankarali H (2009) the effect of the Botulinum toxin-A on craniofacial development: an experimental study. Ann Plast Surg 63(4):449-456.

8. Goodman GJ (2010) The use of botulinum toxin as primary or adjunctive treatment for post acne and traumatic scarring. J Cutan Aesthet Surg 3(2):90-92.

9. Feily A, Fallahi H, Zandian D, Kalantar H (2011) A succinct review of botulinum toxin in dermatology; update of cosmetic and noncosmetic use. J Cosmet Dermatol 10(1):58-67.

10. Gassner HG, Sherris DA, Friedman O (2009) Botulinum toxin-induced immobilization of lower facial wounds. Arch Facial Plast Surg 11(2):140-142.

11. Jablonka EM, Sherris DA, Gassner HG (2012) Botulinum toxin to minimize facial scarring. Facial Plast Surg 28(5):525-535.

12. Laskawi R (2008) The use of botulinum toxin in head and face medicine: an interdisciplinary field. Head Face Med 10; 4:5.

13.Tollefson TT, Senders CM, Sykes JM, Byorth PJ (2006) Botulinum toxin to improve results in cleft lip repair. Arch Facial Plast Surg 8(3):221-222.

14. Flynn TC (2009) Use of intraoperative botulinum toxin in facial reconstruction. Dermatol Surg 35(2):182-188.

15. Shaarawy E, Hegazy RA, Abdel Hay RM (2015) Intralesional botulinum toxin type A equally effective and better tolerated than intralesional steroid in the treatment of keloids: a randomized controlled trial. J Cosmet Dermatol 14(2):161-166.

16. Park TH, Rah DK, Chong Y, Kim JK (2015) The effects of botulinum toxin A on survival of rat TRAM flap with vertical midline scar. Ann Plast Surg 74(1):100-106.

17. Robinson AJ, Khadim MF, Khan K (2013) Keloid scars and treatment with Botulinum Toxin Type A: the Belfast experience. J Plast Reconstr Aesthet Surg 66(3):439-440.
